# Lab-Based Retrospective 10-Year Analysis Shows Seasonal Variation of Vaginal *Candida* Infection Rates in Belgium

**DOI:** 10.3390/jcm11030574

**Published:** 2022-01-24

**Authors:** Gilbert G. G. Donders, Kateryna Ruban, Francesca Donders, Reinhilde Reybrouck

**Affiliations:** 1 Femicare, Clinical Research for Women, 3300 Tienen, Belgium; katerina.s.ruban@gmail.com (K.R.); francesca.donders@gmail.com (F.D.); 2 Department of Obstetrics & Gynecology, Regional Hospital Heilig Hart, Kliniekstraat 45, 3300 Tienen, Belgium; 3 Department of Obstetrics and Gynecology, University Hospital Antwerpen, 2650 Edegem, Belgium; 4 Clinical Biochemistry, Regional Hospital Heilig Hart, 3300 Tienen, Belgium; reinhilde.reybrouck@rztienen.be

**Keywords:** vulvovaginal candidosis, candidiasis, risk factors, management, vitamin D, melatonin

## Abstract

*Candida vulvovaginitis* is a frequent condition, and although several risk factors are known, its behavior is still enigmatic. The seasonal influence of climate conditions and living habits on its prevalence was studied. In a retrospective lab-based cohort over 10 years, we studied the prevalence of *Candida* in 12,941 vaginal cultures taken from women attending a vulvovaginitis clinic. The prevalence of non-albicans and albicans species were compared per month to detect differences in positivity rates in summer versus winter months. Chi-square and chi-square for trend were used. Of the 2109 (16.3%) *Candida* spp. positive swabs, 201 (1.0%) revealed non-albicans species, varying between 1.0% and 2.0% per month, but without significant monthly differences. Over the 10 years, compared to other months, vaginal *Candida* was more frequent in June (19.0%, *p* = 0.008) and less frequent in December (14.5%, *p* = 0.04). The *Candida* prevalence was 15.5% in summer (June/July/August) versus 14.0% in the winter (Dec/Jan/Feb, *p* = 0.04). Change in temperature, dietary habits, and bodily adaptations due to increased amount of sunlight were discussed as potential pathophysiological mechanisms to explain the excess of *Candida* in summertime. Further confirmatory research would be beneficial. Women at risk for *Candida vulvovaginitis* should pay more attention to living habits in summertime to avoid recurrences.

## 1. Introduction

Vulvo-vaginal *Candida* infections are amongst the most frequent reasons to visit a gynecological practice [1,2]. Besides treatment with antifungal medications [3,4,5], several recommendations are given to women to prevent recurrence of such episodes. Although in the 1980s it was suggested that recurrent vaginal *Candida* infections may be caused by sexual activity [6], in recent studies, different types of sexual behavior did not influence the efficiency of maintenance treatment in patients with recurrent vulvovaginal *Candida* infections [7]. Due to more frequent recurrences [8] and increased resistance against antifungal therapy [9] in women with colonization at multiple non-genital sites of their body, spread, especially form the rectum, was considered a possible mechanisms involved in the causation of recurrent vulvovaginal candidosis (RVVC, defined as four or more episodes per year of which at least one is proven by culture), leading to the preference of systemic rather than local therapy for such women [10]. On the other hand, research using rDNA restriction fragment length polymorphisms clearly demonstrated that the vaginal and rectal strains were different and that reinfection due to spread from the anus could not be blamed for recurrences after therapy [11].

Genetic predisposition due to deficient innate immunity is also related to the likelihood of developing recurrent disease, as more women with RVVC are suffering from atopic disease [12,13]. The finding of DNA polymorphisms leading to impaired mannose binding lectin is not only related to more frequent recurrences of VVC [14,15] but is also associated with different response to maintenance therapy [16]. Since decades, intensive research is ongoing to unravel the T-helper 1 and 2 responses to *Candida* infection of the vagina, thereby trying to explain the different responses of women to the same challenge with *Candida* organisms [17,18,19,20,21].

Amongst other mechanisms of pathogenesis, use of antimicrobials, immunosuppression, impaired glucose metabolism, and pregnancy are the most well-known and generally recognized triggering factors [10]. Although recurrences of *Candida* vaginitis are more frequent in diabetic women whose glucose is not well controlled [22,23,24], and some women with recurrent vulvovaginal *Candida* infections tend to have in increased serum glucose level after glucose tolerance testing compared to healthy controls [25], no difference could be detected in response level to fluconazole maintenance therapy in women with RVVC [26]. However, changing the hormonal milieu of the contraceptives and the application of different methods of perineal hygiene are also recognized as helpful means by many women [27]. The latter are most likely linked to the stimulation of *Candida* growth due to the triad of warmth, humidity, and lack of ventilation in the perineal area in women, including the use of perineal pads and tight-fitting clothes [28]. Many of these risk factors are debatable and will vary from practice to practice depending on the own experiences and beliefs.

Temperature, sunlight exposure, dietary changes, and clothing habits may also vary during seasons. In Belgium, summers are typically hot and often humid, winters cold and dry, and intermediate seasons with a great deal of rainfall. Both in summertime and wintertime, even more extreme climate conditions are sought, as people tend to go to even more hot and sunny places in summer and visit ski resorts in winter. Clothing changes from loose and light during summer to warm and multilayered during wintertime. Furthermore, consumption of food and drinks usually varies according to the seasons. As we also had an impression in clinical care that more infections are also present in summer than in winter, we decided to perform a retrospective observational laboratory-based survey to test this hypothesis. Our aim was to detect different infection rates with *Candida* in vaginal swabs in summer or winter months over a 10-year period in women presenting at an outpatient gynecology service in Belgium with vulvovaginal symptoms or follow up of treatment for such symptoms. As a secondary aim, we tested whether non-albicans species could account for any such differences found.

## 2. Methods

### 2.1. Collection of Samples

Vaginal swabs were collected from women presenting at the outpatient gynecology Department of the Regional Hospital Heilig Hart in Tienen, Belgium, from September 2007 until August 2017. Women presented with vulvovaginal complaints or in follow up for infections by gynecologists working at the outpatient clinic. Most swabs originated from women presenting at a specialized vulvo-vaginitis clinic, which was organized 3 times weekly. Patients can be referred by gynecologists or dermatologists, general physicians, or walk in for advice without referral. Seventy percent of patients are from the local community in the central area of Belgium (radius circa 50 km) and are often self-referred, while the remaining 30% originate from more remote areas, mostly referred by their general physician or specialists. Women originating from non-European countries were a minority of 5%, with 30% of them being of Eastern and Southern European origin. At this consultation, mainly patients presenting with recurrent vulvovaginal symptoms, with vague and undetermined symptoms, or for follow up during treatment regimens were seen. The other swabs originated from patients presenting with vulvovaginal symptoms at any consultation throughout the week and at any time of the day. Patients visiting these clinics are largely reimbursed (around 80%) for their consultation fees and almost fully for the lab cultures (95%) by the national general health insurance system. All swabs were taken through a speculum, from the upper lateral vaginal vault.

### 2.2. Laboratory Techniques for Vaginal Candida Cultures

Swabs were immediately transferred into liquid Amies preservation medium (BD CultureSwab™ MaxV(+) Amies Medium without Charcoal (MaxV(+); manufactured by Copan Diagnostics Inc., Murrieta, CA, USA for Becton Dickinson and Co., Sparks, MD, USA) and transported to the laboratory the microbiology laboratory of the Regional Hospital Heilig Hart in Tienen, Belgium at ambient temperature within 2 h to maximal 6 h after sampling. Upon receipt in the laboratory, the swab was inoculated in a tryptic soy agar plate with 5% sheep blood [29]. After 18- to 24-h incubation, the plate was examined for the presence of *Candida*. The results were reported as *C. albicans* if filamentation was seen within two hours when inoculated in human serum. *Candida non-albicans* was subtyped by Vitek II, using the YST card system (BioMérieux, Marcy-l’Étoile, France) [30].

### 2.3. Collection of Seasonal Climate Conditions

Local climate conditions in Belgium, such as the mean amount of sunshine per day (in Kw/h per day), level of clearness (score between 0 and 1), mean number of rainy days, and mean daily temperature per month, were retrieved from the NASA Langley Research Center Atmospheric Science Data Center; New et al., 2002, found at https://www.gaisma.com/en/location/brussels.html (accessed on 12 October 2018).

### 2.4. Statistical Analysis

The proportion of total *Candida* sp., *C. albicans*, and non-albicans positive culture results were reported per accumulated month over the period of 10 years. In case of co-infection of a *C. albicans* and non-albicans strain, it was counted as *C. albicans*.

Statistical analysis was done by chi-square to compare the prevalence during each particular month versus the mean prevalence during the 10-year observation period. A *p*-value of 0.05 was considered significant.

Subsequently, Pearson correlation between the mean monthly percentages of total *Candida*, *C. albicans*, and *C. non-albicans* vaginal infection and the 4 above-mentioned climate characteristics in Belgium during the corresponding month were calculated. The correlation coefficient (R²) and *p*-value are presented. *p*-Values of less than 0.05 were significant.

Finally, the difference between the mean percentages of *Candida* infection during the 3 summer months and 3 winter months were analyzed by the unpaired Student’s *t*-test. These findings were compared with the differences in weather conditions within the same summer and winter months.

## 3. Results

**Figure 1:**
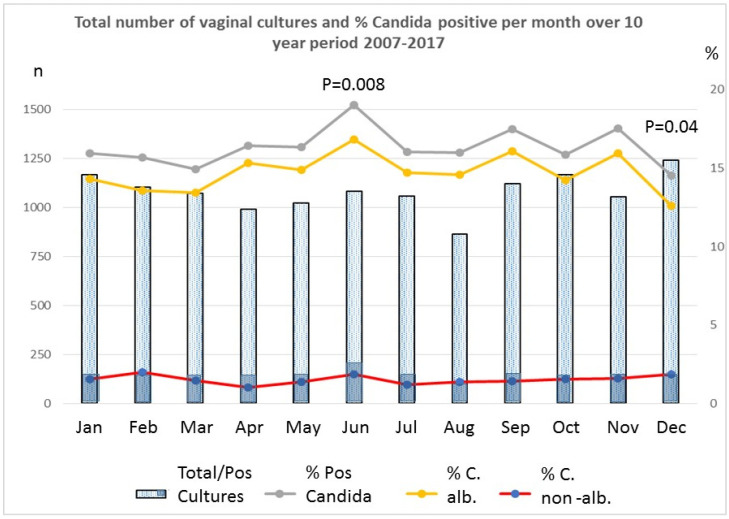
Cumulated monthly total number of vaginal cultures and positive *Candida* cultures received in the Microbiology Laboratory H Hart Hospital Tienen, Belgium, over a 10-year period.

The monthly number of swabs processed during the study period were between 69 and 176, with a mean of 107.8 ± 22.8. As a mean total per month over the 10-year span, the lowest number of cultures were processed in August (*n* = 863) and the most in December (*n* = 1240). Overall, 2109 of 12,941 cultures were positive for *Candida* spp. (16.3%), 201 of which revealed non-albicans species (1.0% of *Candida* positives). The percentage of non-albicans strains varied between 1.0 and 2.0% per month, without significant differences. Over the 10-year period, during the month of June, significantly more cultures were positive for *Candida* sp. (182/1082, 19.0%, *p* = 0.008), while in December, there were less positive *Candida* cultures (180/1240, 14.5%, *p* = 0.04) than in other months (Figure 1). The *Candida* prevalence was 15.5% (948/6136) in the summertime (June/July/August) versus 14.0% (951/6805) in the winter (December/January/February) months (11%, *p* = 0.041). The daily light energy was 4.66 ± 0.30 vs. 0.89 ± 0.35, *p* < 0.0001; clearness score 0.44 ± 0.01 vs. 0.32 ± 0.03, *p* < 0.0001; rainy days 15.2 ± 0,16 vs. 18.13 ± 2.1, *p* < 0.0001; and temperature (°C) 17.69 ± 1.44 vs. 4.13 ± 0.46, *p* < 0.0001 during the same months in summertime and wintertime, respectively.

The correlation between these four climate factors and the percentage of positive *Candida* cultures for the same month is shown in Table 1; although there is a trend towards higher temperatures being linked to a higher prevalence of *Candida albicans* (*p* = 0.07), we failed to link vaginal *Candida* infections to light energy and clarity during the days.

## 4. Discussion

Vaginal cultures studied over a 10-year period in a non-selected population revealed a seasonal variability, with increased rates in summertime, especially in June, and a decreased rate in wintertime (lowest in December). To our best knowledge, this is the first report of this association. Forty years ago, similar seasonal variation was also found in the rates of female genital infections, including vulvovaginitis, although the peak of monilia vaginitis was registered in 1975 later in summer (August–September) than in our series (June) [31]. Increased sexual activity and higher attendance of STI clinics were thought to be responsible at that time. Although some studies seem to indicate a relation between *Candida* vaginitis and sexual activity, suggesting sexual transmission [32], most other studies deny such a relation [33]. Higher attendance could not have accounted for the difference found in our series, as the rate of positivity was reciprocal to the number of visits where cultures were taken (most visits in December, where the positive rate was lowest).

Accordingly, other associations must be responsible for the 11% increase in rate of vaginal presence of *Candida* in summertime. Although highly speculative, the most at-hand explanation could be that people have a different lifestyle in summer. The mean temperature is in Belgium 12 to 16 degrees warmer in summertime (a mean of 3.3–3.9 °C in winter vs. 16.2 to 18.4 °C in summer during 1981–2010 https://www.meteo.be/meteo/view, accessed on 12 October 2018), and many people move to even warmer and sunnier holiday destinations during that period. In this study, we confirmed a link between the frequency of *C. albicans* vaginitis with the mean monthly temperature in the country although this trend was not significant. Higher temperatures may also bring along dietary changes.

Indeed, in previous work, we found women with RVVC to have higher mean serum glucose levels than normal controls [25], and also diabetic women suffer from more frequent and difficult-to-control relapses of RVVC if their glucose is not well regulated [24]. On the other hand, RVVC women not responding to treatment were not more often suffering from (pre-)diabetes and had no higher sugar levels in their serum, urine, or vagina [26], and therefore, this cannot account for a higher number of *Candida*-positive cases in summertime. Therefore, there was a relationship between glucose levels and the presence of RVVC versus normal women, but there was no relationship between treatment response in women with RVVC. Furthermore, older studies did not reveal any seasonal variation in serum glucose levels, insulin levels, body mass index, or starch intake [34,35]. Lipoprotein lipase; serum lipids, such as cholesterol [35]; and fasting glucose and glycosylated hemoglobin were all decreased in summer, again supporting the hypothesis of higher serum glucose levels being responsible for the higher *Candida* rate in summer [34,36].

As melatonin and vitamin D3 increase with increasing hours of daylight [37], an alternative hypothesis could be that these molecules, which have antioxidant and immunomodulatory action, can also be involved in the likelihood to provoke *Candida* infection or vaginitis. Besides sporadic small studies on the effect of melatonin on *Candida* sepsis [38] and phagocytic function of *Candida* cells [39] in animal models or *C. paraspilosis* biofilm formation in vitro [40], there is no evidence of melatonin being involved in the pathogenesis of *Candida* vaginitis in humans. In their study, Ai-Leng Khoo et al. found that increased in-vivo 1.25(OH)_2_D_3_ led to attenuated inflammatory response to *C. albicans* ex-vivo in human volunteers’ [4] modulation of the innate immune response of human leukocytes challenged with *C. albicans*. To validate these findings, peripheral blood mononuclear cells from subjects isolated during each season of the year were stimulated with *C. albicans*, showing a significant drop in inflammatory cytokines (interferon-gamma and interleukin (IL)-17) secretion in spring and summer months, while the anti-inflammatory IL-10 levels were higher in summer as compared to winter [41]. These observations correlated with the serum 25(OH)D_3_ concentrations. In another paper, they showed a physiological increase in vitamin D_3_ storage during summer, leading to down-regulation in pro-inflammatory cytokine production (Il-1β, IL-6, TNF-α) and IL-10, particularly when stimulated via the TLR-4-mediated signaling pathway [42]. Therefore, increased tolerance against *Candida* leading to its increased vaginal infection rates in summer periods could be fully or partly due to decreased innate immunity mediated by increased vitamin D levels from sunlight. In our study, however, we failed to link the tendency to more increased *Candida* vaginitis in summer than in winter to increased clarity or daily light energy during the summer period, making above theory less plausible.

This hypothesis is also in agreement with the observation that vaginal itching and allergic vulvovaginitis may be manifestations of seasonal allergy against pollen [43]. Furthermore, there is a strong association between atopy and RVVC [13], non-response to maintenance therapy in RVVC patients [44], and lack of a strong type 2 immune response to *C. albicans* antigen [13]. In such case, the allergic reaction to other antigens at the vaginal mucosa could facilitate the colonization or infection, with *C. albicans* as a secondary event due to the break of the natural resistance to pathogens. Knowing from other studies that on top of this, type 1 immune response to *C. albicans* is also severely impaired in women with RVVC [45], the observation that RVVC is associated with both atopy and impaired type 1 immune response may have clinical implications. Some authors advocate to combine anti-allergic medication, such as cetirizine, in combination with antifungal treatment for such patients [46].

The advantage of this study is the availability of an unbiased collection of a vast number of clinical samples over 10 years in one single gynecology service, specialized in vulvovaginitis, examined in one single lab over a period of years. The limitations are that no personal information of the patients was available and that we therefore had to rely on a proxy of the climate condition data to link the findings with. Furthermore, we realize that a prospective follow up of these women over 10 years would have been the most ideal scenario, as it would enable us to separate acute infections from women in follow up of some sort of treatment regimen. However, as this is a lab-based analysis using anonymized data, we were not able to elucidate this issue in this retrospective analysis. Finally, standard cultures were performed on vaginal swabs not only to detect *Candida* but also other microorganisms. We realize that the use of specific media, such as Sabouraud, would have yielded higher sensitivity for *Candida* detection. However, we do not think this would interfere with the seasonal relationship described here.

In conclusion, we can confirm for the first time that over a period of 10 years, vaginal *Candida* was more frequent in summer than in the wintertime. Change in temperature, dietary habits, and bodily adaptations due to increased amount of sunlight are potential pathophysiological mechanisms to explain the excess of *Candida* in summertime. Further confirmatory research is needed, as this frequent condition requires deeper investigation. Women at risk for *Candida* vulvovaginitis should pay more attention to living habits in summertime to avoid recurrences.

## Figures and Tables

**Table 1:** Mean percentages of total *Candida*, *C. albicans*, and *C. non-albicans* vaginal infection compared to weather conditions per month in Belgium (NASA Langley Research Center Atmospheric Science Data Center; New et al., 2002. https://www.gaisma.com/en/location/brussels.html, accessed on 12 October 2018).

MONTH	% Tot *Candida*	% *C*. *albicans*	% *C*. Non-*albicans*	Insolation, kWh/m²/Day	Clearness (0–1)	Wet Days	Temp °C
January	15.94	14.31	1.54	0.74	0.32	20	3.77
February	15.67	13.59	1.99	1.37	0.36	15.2	3.84
March	14.91	13.42	1.49	2.46	0.4	18.4	6.11
April	16.45	15.34	1.01	3.72	0.43	16.7	8.72
May	16.36	14.89	1.37	4.77	0.45	16.7	13.04
June	19.04	16.82	1.85	4.89	0.43	15.2	15.87
July	16.05	14.73	1.23	4.85	0.44	15	18.44
August	15.99	14.60	1.39	4.24	0.45	15.4	18.76
September	17.50	16.07	1.43	2.84	0.4	15.3	15.56
October	15.87	14.24	1.54	1.67	0.36	17	12.05
November	17.54	15.92	1.61	0.86	0.31	19.3	7.38
December	14.52	12.58	1.85	0.55	0.29	19.2	4.78
Regression % Total *Candida* infection (r², *p*-value)				R² 0.17, *p* 0.2	R² 0.08, *p* 0.4	R ²0.13, *p* 0.2	R² 0.21, *p* 0.13
Regression % *C. albicans* infection (r², *p*-value)				R² 0.24 *p* 0.1	R² 0.16, *p* 0.2	R² 0.14, *p* 0.2	R² 0.29, *p* 0.07
Regression % *TC. non-albicans* infection (r², *p*-value)				R² 0.23 *p* 0.1	R² 0.30, *p* 0.06	R² 0.01, *p* 0.7	R² 0.16, *p* 0.2

## Data Availability

Full data set is available on request.
